# Temperature and Cyanobacterial Bloom Biomass Influence Phosphorous Cycling in Eutrophic Lake Sediments

**DOI:** 10.1371/journal.pone.0093130

**Published:** 2014-03-28

**Authors:** Mo Chen, Tian-Ran Ye, Lee R. Krumholz, He-Long Jiang

**Affiliations:** 1 State Key Laboratory of Lake Science and Environment, Nanjing Institute of Geography and Limnology, Chinese Academy of Sciences, Nanjing, China; 2 Graduate University of Chinese Academy of Sciences, Beijing, China; 3 Department of Microbiology and Plant Biology, University of Oklahoma, Norman, Oklahoma, United States of America; University of New South Wales, Australia

## Abstract

Cyanobacterial blooms frequently occur in freshwater lakes, subsequently, substantial amounts of decaying cyanobacterial bloom biomass (CBB) settles onto the lake sediments where anaerobic mineralization reactions prevail. Coupled Fe/S cycling processes can influence the mobilization of phosphorus (P) in sediments, with high releases often resulting in eutrophication. To better understand eutrophication in Lake Taihu (PRC), we investigated the effects of CBB and temperature on phosphorus cycling in lake sediments. Results indicated that added CBB not only enhanced sedimentary iron reduction, but also resulted in a change from net sulfur oxidation to sulfate reduction, which jointly resulted in a spike of soluble Fe(II) and the formation of FeS/FeS_2_. Phosphate release was also enhanced with CBB amendment along with increases in reduced sulfur. Further release of phosphate was associated with increases in incubation temperature. In addition, CBB amendment resulted in a shift in P from the Fe-adsorbed P and the relatively unreactive Residual-P pools to the more reactive Al-adsorbed P, Ca-bound P and organic-P pools. Phosphorus cycling rates increased on addition of CBB and were higher at elevated temperatures, resulting in increased phosphorus release from sediments. These findings suggest that settling of CBB into sediments will likely increase the extent of eutrophication in aquatic environments and these processes will be magnified at higher temperatures.

## Introduction

Due to climate change and anthropogenic carbon and nitrogen runoff, cyanobacterial blooms are becoming more common in freshwater lakes and estuaries throughout the world, threatening the sustainability of aquatic ecosystems [1], [2]. The formation of large mucilaginous cyanobacterial blooms in freshwater lakes restricts light penetration, which depletes oxygen levels, thereby reducing water quality and adversely affecting the ecosystem [1]. These changes can result in reduction in the numbers of submerged plants, death of aquatic animals, and alteration of food web dynamics [3].

As cyanobacterial bloom biomass (CBB) dies, it settles on surface sediments, eventually becoming incorporated into sediments through resuspension and bioturbation. Decomposed CBB can be an important benthic food source [4] and decomposition products can be assimilated by rooted macrophytes [5]. As CBB undergoes decomposition, both nitrogen and phosphorus containing compounds are released. This release results in changes in the nutrient composition of sediments and water and eventually alters the sediment microbial community [6]. While decaying CBB in sediments has been found to strongly influence the bacterial community composition of lake sediments [7], the role of settled CBB in biogeochemical cycling in lakes with seasonal temperature changes have not been well studied.

In lakes, especially shallow lakes, sediment processes dominate the overall metabolic activities [8]. Lake sediments are important in the global carbon cycle, as they can act as both a sink and a source of critical elements [9]. Phosphorous is cycled in lake sediments [10] and excessive phosphorous input often directly causes eutrophication [11]. Phosphorus occurs in lake sediments in both organic and inorganic forms. Inorganic phosphorus typically associates with amorphous and crystalline forms of Fe, Al, Ca, and other elements. Organic phosphorus varies in ease of decomposition, therefore in phosphate bioavailability [12]. Transformation of phosphate compounds in sediments is highly dependent on environmental parameters, with the most important being temperature and redox potential. Typically, an increase in temperature depletes labile organic phosphorus [13]. Also, internal phosphorus cycling in sediments can be enhanced by increased temperatures, leading to more significant eutrophication [14]. Although increased temperatures result in higher levels of P release [15], especially in concert with extended anoxic conditions [16], a substantial fraction of P still remains buried in sediments. The mechanisms behind these shifts in P pools, as well as the impact of seasonal temperature changes and settled CBB on P movement between pools under anaerobic conditions, has not been well studied. Phosphorus cycling is also intertwined with iron and sulfur cycling. Under anaerobic conditions, both iron and sulfate reduction increase phosphorus release from sediments [17]. The lowering of the redox potential allows reduction of Fe (III) in iron oxides to Fe(II), resulting in a decrease in Fe-bound phosphorus [18].

In this study, the effect of settled CBB and seasonal temperature changes on phosphorus cycling in the sediments of a subtropical shallow lake were investigated. CBB was amended into surface sediments taken from a eutrophic lake, Lake Taihu, and sediments were incubated under anaerobic conditions at temperatures ranging from 4 to 32°C. Our results indicate that settling of CBB into sediments and increasing temperatures likely play important roles in influencing Fe/S biogeochemical processes and maintaining the eutrophic status of lakes.

## Materials and Methods

### Ethics statement

No specific permits were required for the described field studies. The location studied is not privately-owned or protected in any way and our studies did not involve any endangered or protected species.

### Sediment sampling

Samples of both sediments and CBB were taken from Lake Taihu. Lake Taihu (31°10′ N, 120°24′ E), the third largest shallow freshwater lake in China, is situated south of the Yangtze River delta. It has a water surface area of 2340 km^2^ and mean and maximum depths of 1.9 m and 3.4 m, respectively [19]. Water temperatures are between 0 and 38.0°C, with minimum temperatures occurring in January and maxima in July and August [20]. Increasing nutrient inputs associated with both population and economic growth have led to eutrophication in the lake. The average total phosphorus and sulfate concentrations in lake water are 0.086 and 100 mg L^−1^, respectively. Since the 1980s, cyanobacterial blooms have occurred with increasing frequency and intensity in Lake Taihu [21].

Sediments were sampled using a gravity core sampler, and CBB samples were harvested by sieving lake surface water through a fine mesh plankton net in May, 2012. CBB samples were immediately stored in polyethylene bottles. Sediments and CBB samples were placed on ice and transported to the laboratory within several hours of collection. Subsequent storage of all samples was at 4°C for less than 24 hours until usage.

### Sediment incubations

CBB samples were poured in to medical trays and placed in a Fume hood for several days to air dry. The dried CBB was then scraped into a mortar and ground into powder. Surface sediments (0–5 cm) from 18 sediments cores (10-cm-i.d. Plexiglas tubes) were sliced and homogenized thoroughly in a nitrogen-filled glove bag. Sediments with wet weights of 300 g and air-dried CBB with weights of 2 g were mixed thoroughly in a 500-mL glass jar inside the nitrogen-filled glove bag. Unamended experiments did not contain CBB. Finally, the glass jars containing sediment and CBB mixtures or unamended sediment were sealed tightly with rubber stoppers under pure nitrogen and incubated at 4°C, 15°C, 25°C or 32°C in a dark environment. Sediments were incubated for 41 days. During this period, the glass jars were opened in the nitrogen-filled glove bag for sampling every 2–13 days. Immediately after sampling, the jars were sealed tightly and incubation was resumed. The initial water content in unamended and bloom-amended sediments were 51% and 50.6%, respectively. Experiments were performed in triplicate.

### Determination of iron and sulfate reduction rates

Fe (III) reduction rates were calculated after Fe (II) concentration in the sediments was determined using the method described below. Rates were calculated by regression of Fe (II) concentration vs. time [22]. Similarly, sulfate reduction rates were calculated based on regressions of sulfate concentrations vs. time.

### Analytical methods

Water content in sediments was determined as weight loss after drying at 105°C for 12 hours. Total organic carbon (TOC) in sediments was measured with Walkley and Black's rapid titration method [23]. pH in sediments was measured by inserting a glass electrode calibrated with NBS standards (National Bureau of Standards, Gaithersburg, USA) directly into sediments.

Total phosphorus (TP) content in sediments was analyzed as phosphate after acid hydrolysis at 340°C [24]. The presence of different forms of phosphorus was determined through sequential extractions and digestions [25]. Samples were air-dried and extracted sequentially as follows: (a) 1 M NH_4_Cl at pH 7, (b) 0.11 M NaHCO_3_/0.11 M Na_2_S_2_O_4_, (c) 0.1 M NaOH, and (d) 0.5 M HCl. The different forms of phosphorus extracted according to the above processes were referred to as (with description in parentheses): (a) NH_4_Cl-P (Loosely bound or labile-P including soluble reactive phosphate (SRP) in pore water, Labile-P); (b) Fe-adsorbed P (Fe-P); (c) NaOH-rP (Al-adsorbed P, Al-P). (d) HCl-P (Ca-bound P, Ca-P); (e) Residual-P (residual P, Res-P). Res-P was calculated as TP (determined above using acid hydrolysis) minus the total phosphorus measured during the sequential extraction. Organic P (Org-P) was calculated from the difference between total NaOH extracted P (NaOH-Tot P) and NaOH-rP (Al-P) after digestion in the NaOH extraction step c [25].

To determine the dissolved Fe(II) concentration, sediment samples were placed into a 2 mL centrifuge tube in the nitrogen-filled glove bag, leaving no gas phase. The sediment samples were centrifuged at 12000 rpm for 30 sec and 0.1 mL of the supernatant was acidified with 0.1 mL of 1 M HCl (under pure nitrogen). Concentrations of Fe(II) was determined colorimetrically [26]. Sulfate and orthophosphate (PO_4_^3−^) concentrations in pore water were measured using ion chromatography (ICS-2000, Dionex, USA).Sediment Amorphous Fe(III) oxide and total Fe(II) concentrations in the sediments were measured by extracting sediments with 0.5 M HCl, followed by determination using the Ferrozine method [26]. Total Fe was reduced with hydroxylamine hydrochloride and Fe(III) concentration was calculated from the difference between total Fe and Fe (II).

Concentrations of acid volatile sulfide (AVS) and chromium reducible sulphur (CRS) were determined according to the procedure described previously [27], with slight modifications. Briefly, 2 g wet sediment was put into a sample flask with a small vial containing 5 mL alkaline Zn solution. The flask was closed with a two-outlet stopper and flushed with nitrogen gas for 5 min, then both outlets of the flask were closed. In order to measure AVS (including dissolved sulfide and FeS), 15 mL of deoxygenated 9 M HCl solution and 2 mL of 1 M ascorbic acid solution were injected into the flask through the syringe stopcock in a nitrogen filled glove bag. The syringe stopcock was closed, and the flask was incubated at room temperature. The flask was opened after 24 hours and the Zn trap retrieved to determine the concentration of precipitated Zn-sulfide. Immediately after the Zn trap was retrieved, the trap (5 mL alkaline Zn solution) was replaced and the flask closed and flushed with nitrogen again for the following CRS (pyrite-S) separation. Cr(II) solution (15 mL) was immediately injected into the flask from the previous AVS procedure, after replacing the small vial containing 5 mL fresh alkaline Zn solution. The sample flask was then closed, allowing the reaction to take place at room temperature. After 48 hours, the flask was opened and the Zn trap was retrieved for Zn-sulfide analysis as described above.

The temperature coefficient (Q10) can be used to express the influence of temperature on the rate of various metabolic processes [28]. In this study, Q10 was used to investigate the influence of temperature on the rate of iron reduction, sulfate reduction, and phosphate release in sediments. Q10 values were calculated from reduction and release rates at 15, 25 and 32°C, using the equation: 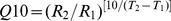, where R_2_ and R_1_ are the metabolic rates at two temperatures, T_2_ and T_1_.

### Statistical analyses

Statistical significance of differences was determined by one-way analysis of variance using Origin Pro 7.5 and SPSS 19.0 software. A *P*<0.05 was considered significant.

## Results

### Concentrations of inorganic elements (Fe, S, and P) in pore-water

**Figure 1:**
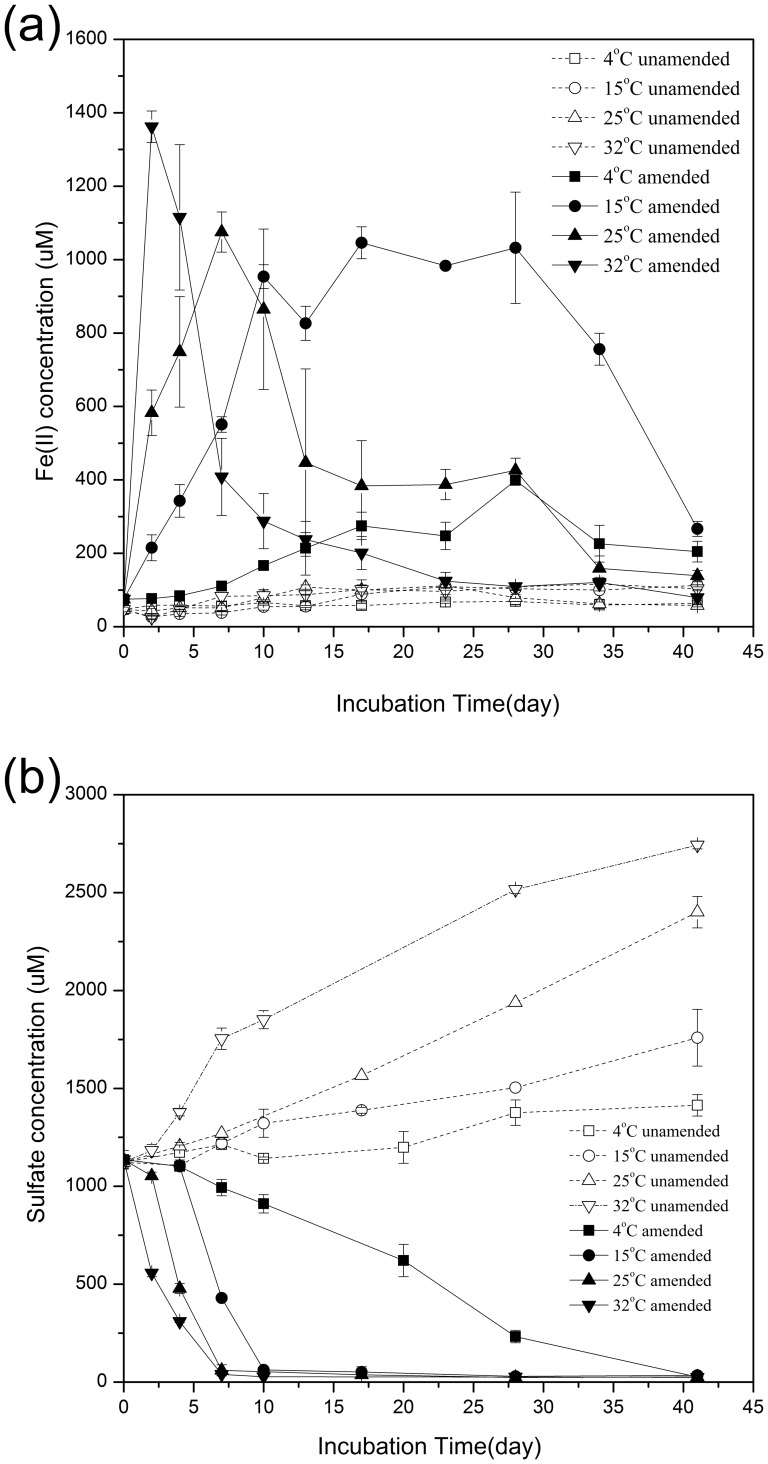
Fe(II) concentrations (a), and sulfate concentrations in sediment pore-water (b).

The initial concentrations of Fe(II) in pore-water in unamended and CBB-amended sediments were 47.0±6.4 μmol L^−1^ and 75.1±6.2 μmol L^−1^, respectively (Fig. 1a). Fe(II) concentration in the pore-water of CBB-amended sediments at 32°C increased rapidly to a maximum of 1361.8±43.1 μmol L^−1^ on day 2 (Fig. 1a) and then decreased quickly over the next 5 days. The maximum concentration of Fe(II) in the pore-water in CBB-amended sediments at 4°C (398.7±5.1 μmol L^−1^), 15°C (1045.9±43.4 μmol L^−1^) and 25°C (1075.1±54.5 μmol L^−1^) appeared on days 28, 17, and 7, respectively (Fig. 1a). In unamended sediments, Fe(II) concentration in pore-water increased to around 100 μmol L^−1^ at higher temperatures (15°C, 25°C, and 32°C), compared to no obvious change of Fe(II) concentration at 4°C (Fig. 1a).

Initial sulfate concentration in the pore-water of unamended and CBB-amended sediments was around 1122–1136 μmol L^−1^. As shown in Fig. 1b, in sediments without CBB amendment, the sulfate concentration in pore-water increased gradually at 15°C, 25°C and 32°C, with little change at 4°C. Rate of sulfate production was a function of the increase in temperature. In CBB-amended incubations at 32°C, sulfate reduction occurred immediately. Sulfate concentration decreased during the first 7 days and stayed at approximately 26 μmol L^−1^ until the end of the experiment (Fig. 1b). The rate of sulfate reduction was also temperature dependent, with relatively rapid loss of sulfate at 25°C and a marked lag in sulfate reduction at the other two temperatures (4°C and 15°C). At 4°C, sulfate concentration declined slowly until day 41, finally reaching a level similar to those seen in other experiments.

**Figure 2:**
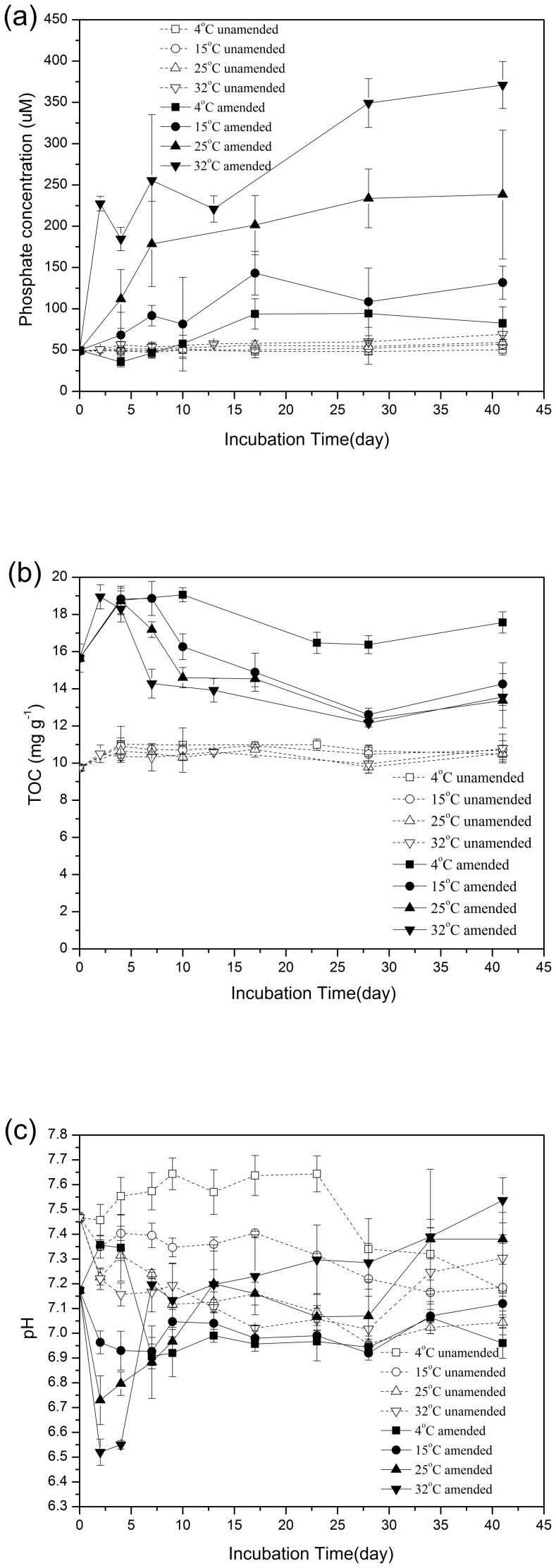
Phosphate concentrations in sediment pore-water(a), total organic carbon (TOC)(b) and pH in sediments(c).

Orthophosphate concentration in pore-water of unamended sediments had an initial concentration of 48.8±4.6 μmol L^−1^ and increased only slightly, reaching peak concentrations of 50.6±6.5 μmol L^−1^ at 4°C, 56.8±5.8 μmol L^−1^ at 15°C, 59.3±8.5 μmol L^−1^ at 25°C, and 69.0±8.6 μmol L^−1^ at 32°C (Fig. 2a). In contrast, CBB amendment resulted in higher pore-water phosphate concentrations at all incubation temperatures. Also, higher incubation temperature was associated with higher phosphate concentrations in CBB amended sediments (Fig. 2a). Maximum phosphate concentrations were 82.8±19.7 μmol L^−1^ at 4°C, 131.8±20.0 μmol L^−1^ at 15°C, 238.5±78.0 μmol L^−1^ at 25°C, and 371.0±28.4 μmol L^−1^ at 32°C. The effect of CBB amendment on phosphate release was significant at all temperatures (*P* = 0.024 at 4°C, 0.022 at 15°C, 0.017 at 25°C, and <0.001 at 32°C).

### Iron and sulfate reduction in sediments

The initial Fe(II) levels in unamended and CBB-amended sediments were 27.9±3.1 μmol g^−1^ and 27.1±2.4 μmol g^−1^, respectively. Peak Fe(II) concentrations ranged from 34.1±1.6 μmol g^−1^ to 59.8±7.3 μmol g^−1^ in unamended sediments and from 40.3±5.2 μmol g^−1^ to 74.6±2.3 μmol g^−1^ in the CBB-amended sediments. Peak concentrations increased with incubation temperature (Table 1). The rate of iron reduction varied from 1.96±1.72 μmol cm^−3^ day^−1^ to 4.51±0.81 μmol cm^−3^ day^−1^ in the unamended sediments and from 5.93±4.00 μmol cm^−3^ day^−1^ to 18.31±2.29 μmol cm^−3^ day^−1^ in the CBB-amended sediments with increasing temperatures from 15 to 32°C. No obvious iron reduction occurred at 4°C in either system.

**Table 1:** Iron reduction and sulfate reduction in sediments.

	15°C		25°C		32°C	
	Unamended CBB-amended		Unamended CBB-amended		Unamended CBB-amended	
Iron reduction rate (μmol cm^−3^ day^−1^)	1.96±1.72△	5.93±4.00^b^	3.00±0.46^a^	8.85±2.48^c^	4.51±0.8^a^	18.31±2.29^b^^,^ c
Maximum Fe(II) concentration (μmol cm^−3^)	65.88±9.10 (17)¶	90.67±15.88 (34)	68.77±4.25 (10)	96.65±4.72 (10)	86.32±10.52 (10)	107.74±3.37 (4)
Sulfate reduction rate (μmol cm^−3^ day^−1^)^d^	ND*	0.085±0.001	ND	0.122±0.006	ND	0.152±0.008

△ Data are means ± standard deviation;

¶ Parentheses indicate the time (day) of the maximum iron reduction rate;

*ND, not detected;

a Significant difference for iron reduction at 25°C and 32°C in unamended sediments (*P*<0.05);

b Significant difference for iron reduction at 15°C and 32°C in CBB-amended sediments (*P*<0.05);

c Significant difference for iron reduction at 25°C and 32°C in CBB-amended sediments (*P*<0.05);

d Sulfate reduction rates in amended sediments at any two respective temperatures show significant difference (*P*<0.05).

The CBB-amendment experiments were carried out at four temperatures. Thus, it was possible to calculate the temperature dependence of metabolic processes, which were expressed as Q10 values. Q10 values of iron reduction ranged from 1.19 to 3.54 in CBB-amended incubations based on paired comparisons (Table 2). Interestingly, the highest Q10 value in amended experiments was 3.54, with incubation temperature increasing from 25°C to 32°C, indicating that the rate of iron reduction with CBB amendment increased most at higher temperatures.

**Table 2:** Temperature effects on calculated Q10 of metabolic processes in freshwater systems.

Temperature interval	Iron reduction	Sulfate reduction	Phosphate release	Sample source	Reference	Trophic status
15–25°C	1.19	1.44	1.19	Lake Taihu	This study	Hyper-eutrophic
25–32°C	3.54	1.37	1.61	Lake Taihu	This study	Hyper-eutrophic
5–15°C		2.25		Lake Constance	[57]	Meso-eutrophic
6.3–20°C		1.07*		Lake Kizaki	[58]	Meso-eutrophic
20–30°C		1.61*		Lake Kizaki		
4–37°C		2.9		Lake Mendota	[59]	Eutrophic
15–25°C			1.03	Cootes Paradise Marsh	[60]	
10–20°C			1.21	Cootes Paradise Marsh		
12–19°C			1.9	Lake Arreskov Littoral sediment	[61]	Eutrophic
20–35°C			1.18	SP-1,Jamsil Dam area,Han River	[62]	

*Q10 values were calculated from the data in the reference.

While sulfate reduction did not occur in unamended sediments, the rates of sulfate reduction calculated using pore-water sulfate concentrations in the CBB-amended experiments ranged from 0.017±0.001 μmol cm^−3^ day^−1^ at 4°C to 0.152±0.008 μmol cm^−3^ day^−1^ at 32°C (Table 1). Rate of sulfate reduction increased linearly with temperature, with Q10 values ranging from 1.37 to 1.44 in the CBB-amended incubation (Table 2). Q10 values for phosphorus release rates ranged from 1.19 to 1.61 in the CBB-amended incubation, with the highest Q10 value occuring as temperature increased from 25°C to 32°C.

### AVS and CRS in sediments

The initial value of AVS in unamended and bloom-amended experiments was about 0.6 μg g^−1^ with initial CRS values of 41.5±0.8 and 41.8±1.1 μg g^−1^, respectively (Table 3). For unamended sediments, AVS and CRS values did not change much over the entire incubation at all temperatures. However, at the end of the experiments, AVS and CRS values in the CBB-amended sediments had increased significantly. They also became higher with increasing incubation temperature, suggesting that the higher levels of reduced iron and sulfur may interact to form more FeS/FeS_2_.

**Table 3:** Acid volatile sulfide (AVS) and chromium reducible sulphur (CRS) content in sediments.

			Final values at the end of experiments (μg g^−1^)							
	Initial values (μg g^−1^)		4°C		15°C		25°C		32°C	
	AVS	CRS	AVS	CRS	AVS	CRS	AVS	CRS	AVS	CRS
Unamended	0.6±0.1△	41.5±0.8	1.0±0.2	41.1±3.9	0.9±0.2	43.9±3.0	0.7±0.0	40.2±1.8	1.0±0.0	41.7±3.0
CBB-amended	0.6±0.0	41.8±1.1	1.7±0.2^a,b,c^	50.9*±4.7^A,B^	10.9*±0.3^a,d^	55.3*±3.1^C^	11.0*±0.3^b,e^	65.7*±3.9^A,D^	13.2*±0.3^c,d,e^	72.1*±1.0^B,C,D^

△ Data are means ± standard deviation.

*Significant differences between initial values and final values at the respective temperatures (*P*<°.05).

a–e The same letter represents a significant difference for AVS at two different temperatures (*P*<0.05).

A–D The same letter represents a significant difference for CRS at two different temperatures (*P*<0.05).

### TOC and pH in sediments

TOC content of sediments was measured and is shown in Fig. 2b. In the unamended sediments, the initial average TOC was 9.7 mg g^−1^. TOC levels in sediments did not change much during the incubation (Fig. 2b). For the CBB-amended sediments with an initial average level of TOC of 15.7 mg g^−1^ (Fig. 2b), TOC in sediments increased slightly to around 18.5 mg g^−1^ during the initial four days at all incubation temperatures. Thereafter, TOC levels decreased at 25°C and 32°C within several days, and then fluctuated around 12.5–14 mg g^−1^ until the end of the experiments. The increase in TOC was likely due to the procedure measuring relatively labile organic carbon more effectively than more refractory compounds [29]. The TOC increase suggests that added bloom biomass enhanced transformation of some of the refractory organic matter or that bloom biomass might contain some refractory compounds.

At 25°C and 32°C, pH values in the CBB-amended sediments first rapidly declined, possibly due to biomass fermentation in the initial of two days, and then increased (Fig. 2c). At the end of the experiments, only pH values in the CBB-amended sediments at 25°C and 32°C were higher than initial pH values in sediments, reaching 7.38±0.02 and 7.54±0.09, respectively (Fig. 2c). The decrease that initially occurred in all sediments was likely associated with production of fermentation products and the subsequent increase in pH may result from iron reduction and sulfate reduction, which can consume protons and fermentation products.

### Phosphate fractionation

The initial TP in CBB-amended sediments was 880.7±5.5 μg g^−1^, only slightly higher than that in the unamended sediments (844.0±2.7 μg g^−1^) (Table 4). Labile-P levels were low (5–8 μg g^−1^) and did not show much variation throughout the experiment. The decrease in Fe-P content was greater at higher temperatures in both amended and unamended experiments, and was also affected by the CBB-amendment. Final values of Fe-P decreased by as much as 13.3% in the unamended sediments and 54.5% in CBB-amended sediments during the incubation (Table 4). The decrease in Fe-P was associated with higher levels of iron reduction, with the greatest change in sediments with amendment of bloom biomass. Al-P levels decreased slightly in unamended sediments and high incubation temperatures led to greater reduction in Al-P. In contrast, Al-P levels in the CBB-amended sediments was observed to increase, and concentrations increased more with higher incubation temperatures.

**Table 4:** Changes in concentrations in individual phosphorous pools during sediment incubations.

			Final values at the end of experiments (μg g^−1^)							
	Initial value (μg g^−1^)		4°C		15°C		25°C		32°C	
	Unamended Amended		Unamended Amended		Unamended Amended		Unamended Amended		Unamended Amended	
Labile-P	5.6±0.2△	5.5±0.3	7.4±2.0	6.1±0.3	6.1±0.6	4.1±1.9	7.0±0.3	5.7±0.8	7.6±0.4	7.5±4.5
Fe-P	173.3±4.6	200.0±3.4	178.8±6.1	187.8±17.2^A^	174.1±17.2	132.5*±17.1	166.7±9.2	130.9*±40.8	150.3*±7.3	91.0*±3.5^A^
Al-P	148.3±1.4	155.6±6.9	142.7±0.9	152.0±11.3^B,C,D^	136.7±0.0	208.3*±2.3^B,E^	133.9*±15.0	200.7*±22.6^C,F^	120.5*±0.8	269.0*±8.0^D,E,F^
Ca-P	151.1±2.5	158.7±5.5	173.6*±21.2	170.8±0.3	165.8*±8.2	183.3*±2.8	170.3*±7.0	207.1*±12.6	178.5*±3.5	203.1*±30.7
Org-P	60.5±1.2	71.3±9.9	93.2*±6.4^a^	116.1*±18.7	98.2*±4.1^b^	108.1*±18.6	97.6*±6.3^c^	141.9*±25.9	115.0*±6.7^a,b,c^	151.4*±36.8
Res-P	305.2±3.0	289.7±6.7	248.6*±2.8^d,e^	247.8*±6.5^G,H^	257.1*±4.3	245.4*±0.4^I,J^	269.6*±4.5^d^	197.5*±0.7^G,I,K^	269.2*±6.1^e^	160.8*±1.1^H,J,K^
TP	844.0±2.7	880.7±5.5	844.4±25.4	880.7±31.6	838.0±25.8	881.7±32.9	845.0± 14.8	883.7±31.5	841.1±15.6	882.7±3.9

△ Data are means ± standard deviation.

*Significant differences between initial values and final values at the respective temperatures (*P*<°.05).

a–e The same letter represents a significant difference at two different temperatures in unamended sediments (*P*<0.05).

A–K The same letter represents a significant difference at two different temperatures in CBB-amended sediments (*P*<0.05)

Both Ca-P and organic-P concentrations increased during incubation of unamended and amended sediments with resulting increases of 12.2 to 48.4 μg g^−1^. However, Ca-P levels were less sensitive to temperature and CBB amendment than other pools. In contrast, the organic-P content of bloom-amended sediments at the end of experiments was much higher than that in unamended experiments at all incubation temperatures. Temperature had a lesser effect on levels of organic-P, with increased levels only observed after the 32°C CBB unamended incubation (Table 4).

At the end of the experiments, Res-P contents of the sediments were less than the initial values (Table 4). Res-P content in unamended sediments did not vary much at the four incubation temperatures, and decreased by about 45 μg g^−1^ by the end of the experiments from an initial value of 305.2±3.0 μg g^−1^. For biomass-amended sediments, Res-P content decreased as a function of increase in temperature, ranging from 41.9 μg g^−1^ at 4°C to 128.9 μg g^−1^ at 32°C. Phosphate moved from the Fe-P and Res-P pools to the Al-P, Ca-P and organic-P pools in the amended sediments and from the Fe-P, Al-P and Res-P pools to the Ca-P and organic-P pools in the unamended sediments.

Based on our statistical analyses, CBB amendment had a significant influence on all P pools except Labile-P. Fe-P, Al-P, and Res-P levels were significantly different between temperature intervals (*P*<0.05), indicating that increased temperatures play an important role in regulating P conversions following an influx of CBB. Organic P levels were unexpectedly not significantly influenced by temperature in CBB amended samples(*P*>0.05). However, levels of error were high for these analyses. In unamended samples, only organic P and Res-P pools were significantly influenced by temperature (*P*<0.05), indicating organic P and Res-P pools are the most temperature-sensitive pools. Moreover, CBB not only enhanced P pool cycling and conversion but also had the most significant impact on concentrations in Fe-P and Al-P pools.

## Discussion

In shallow lakes, the majority of metabolic activities, including most of the organic matter mineralization and nutrient cycling, occur in sediments [8]. Here we report that CBB addition strongly influences biogeochemical processes in sediments taken from the shallow Lake Taihu. Aside from the obvious increase in rate and extent of iron reduction, CBB addition to sediments reversed the sulfur transformation process from sulfur oxidation to sulfate reduction.

Results show that TOC levels in unamended sediments did not change much at any temperature (Fig. 2b), suggesting that organic matter in sediments was somewhat resistant to microbial utilization. Under these conditions, the availability of electron acceptors allowed chemoautotrophic processes to proceed, specifically the anaerobic oxidation of reduced sulfur compounds [30], which resulted in increasing sulfate concentrations. With CBB addition, sulfate reduction occurred and sulfides accumulated. This enhancement of sulfate reduction then affected iron cycling in sediments, as sulfides reduce Fe(III) directly and precipitate Fe(II), resulting in the removal of soluble Fe(II) and the formation and burial of FeS and pyrite [31]. CBB amendment directly increased the Fe loading in sediments (Table 3). Typically, Fe(II) forms ferrous phosphate compounds such as vivianite (Fe_3_(PO_4_)_2_·8H_2_O) [32], [33], however, when Fe(II) is removed from the system by precipitation, phosphate is released into pore water or the overlying waters. Coupled Fe/S cycles after CBB addition to sediments, directly affected the mobilization of phosphorus (Fig. 2a).

While Fe/S cycling, including dissolution of Fe(III) phases and precipitation of FeS/FeS_2_, is regarded as a critical step in phosphorus release from estuarine and marine sediments [17], [34], standing pools of sulfate are typically in the low micromolar-range in lake sediments and are generally interpreted to be too small for sustained levels of sulfate reduction. Therefore, previous work assumed that direct reduction of FeOOH in freshwater sediments was predominately responsible for phosphorus release [35]. Our work indicates that the role of sulfate reduction in the biogeochemistry of freshwater ecosystems has likely been underestimated [36].

Recently, human practices, including the combustion of coal and other fossil fuels and the application of agricultural fertilizer, have increased sulfate input into aquatic ecosystems [37]. Thus, the impact of sulfate input is increasingly being seen as an issue in the management of inland aquatic ecosystems. Cumulative evidence from low sulfate lakes supports the presence of a hidden sulfur cycle [38]. Currently, sulfate levels in Lake Taihu are much higher than those typically observed in freshwater lakes [39], reaching about 1 mM. The effect of this high level of sulfate and the role of the sulfur cycle has not been previously considered in eutrophic freshwater lakes like Lake Taihu. Previously, the roles of Fe, phosphorus, and nitrogen were emphasized [11], [40]. This study indicates that sulfate transformations must be considered, especially when a large organic input occurs into lake sediments. Sulfate reduction, increasing water temperature, settled CBB, and high sulfate concentrations all dramatically increase the release of P in sediments, which can allow development of algal blooms and other serious eutrophication problems.

Also, amended CBB had an impact on the fractionation of phosphorus in sediments, with increases in Al-P, Ca-P, and organic P pools and decreases in Fe-P and the Residual-P pools. The Fe-P pool was reduced as a result of Fe(III) reduction and subsequent dissolution of Fe compounds. The greatest increase was seen in the Al-P pool after CBB amendment. Previous work has reported that Fe(III) reduction caused a transformation of Fe-P to Al-P [25]. In this study, variation in Al-P levels in sediments might be partially due to variation in sediment pH. Decline in pH during the initial period was likely due to decomposition of algae, which could lead to the dissolution of Al compounds in sediments and the release of soluble Al species [41]. Al binds phosphate most effectively under weakly alkaline conditions [42]. Also, recently formed Al oxyhydroxides bind much more orthophosphate than their diagenetically altered forms [41]. Another pool that was increased during this study was Ca-P (Table 4). Other groups have observed the transformation of Fe-P to Ca-P [43]. In comparison to Fe-P, Ca-P and Al-P are not sensitive to redox changes. Thus, incremental increases in Al-P and Ca-P lead to an increase in the level of phosphorus burial and a lower risk of phosphorus mobilization from sediments [44].

However, transformation of other forms of phosphorus also needs to be considered when evaluating phosphorus burial. The organic-P pool was observed to increase in all CBB amended samples (Table 4). Although release of phosphate from organic P likely occurs during CBB decomposition, the addition of labile organic matter such as CBB can stimulate microbial growth and anaerobic metabolism in sediments [45]. Microorganisms can then incorporate dissolved inorganic phosphorus into the cellular constituents and synthesize organic-P compounds such as phosphonate monoesters and phospholipids [46]. Increased organic P burial due to enhanced burial of organic matter has been previously observed [47].

Under normal conditions, the majority of organic-P in surface sediments in Lake Taihu is stable [48]. But, following organic input, an increase in organic P in sediments can occur (Table 4), accompanied by a further increase as a result of the input of cyanbacterial biomass formed during eutrophication. This increase in organic P indicates that bioavailable organic matter may maintain the eutrophic status of lakes after external sources of P have been controlled [49]. Others have also argued that in shallow lakes, an infusion of oxygen caused by sediment resuspension could result in rapid mineralization of organic P, further exacerbating eutrophication [50].

As with Fe-P, the Res-P pool decreased significantly in CBB amended sediments (Table 4). Although biotic or abiotic transformations of Res-P are possible and depend on a range of environmental factors, Res-P is generally regarded as nonreactive phosphorus [51]. The conversion of Res-P to more reactive forms of P as a result of the CBB amendment could also lead to phosphorous release and affect pathways of P flow. This effect on P regeneration and burial in lakes has not previously been investigated, and should be considered in restoration of lake ecosystems.

Many factors influence phosphorus pools in sediments, such as sediment resuspension, alum application, sediment microorganisms, *E*_h_, and pH [25], [42], [52], [53]. In this study, we focused on the influence of CBB and temperature on phosphorus pools. Results indicated that phosphate moved from the Fe-P and Res-P pools to Al-P, Ca-P, and organic-P in the amended sediments and from Fe-P, Al-P, and Res-P pools to Ca-P and organic-P pools in the unamended sediments (Table 4, Figure S1).

Global surface temperature has increased by about 0.2°C per decade in the past 30 years [54], with major increases (0.55°C) occurring during that time. These increases will likely influence iron, sulfur, and phosphorus cycling in aquatic ecosystems [15], [55], [56]. Our Q10 values indicate that temperature increases have the greatest effect on iron reduction, with a more linear effect on phosphate release and sulfate reduction (Table 2). Climate change affects biogeochemical cycling in lakes and the results presented here indicate that iron reduction will likely increase in importance relative to sulfate reduction as temperatures warm. When Q10 data from this work is compared to other, mainly eutrophic freshwater systems (Table 2), similar results are observed, indicating that this data for phosphorous release and the effects of increasing temperatures could likely be extrapolated to other systems. The dramatic effect of temperature on iron reduction between 25 and 32°C indicates the significant effect that climate change could have on the system and the potential for further phosphate release as a result of warming. Along with their impact on individual processes, higher temperatures have important effects on the cycling of P between pools, resulting in increases insoluble P, Al-P, Ca-P, and organic-P. Therefore, higher temperatures might change the pathways of P flow and lead to increased amounts of phosphorous release due to direct increases in rates of release processes as well as effects on the levels of CBB production. Ultimately, the result will likely be the maintenance of the eutrophic status of the system.

## Conclusion

The CBB amendment in sediments not only enhanced iron reduction, but also reversed sulfur oxidation to sulfate reduction, which jointly led to the spike in Fe in the sediments. Coupled Fe/S cycling further led to the mobilization of phosphorus in sediments. Considering the critical role of sulfate reduction in phosphorus release from sediments, special attention should be paid to sulfate pollution in freshwater lakes. The amount of phosphorus released from sediments increased after amending CBB, especially at higher temperatures. CBB amendment at high temperatures also caused a shift from Fe-P and the Res-P pools to Al-P, Ca-P, and organic-P in the sediments. The decrease in inert Res-P in sediments led to an increase in levels of more reactive phosphorus. Therefore, settling of cyanobacterial bloom biomass strongly influences Fe/S biogeochemical processes, specifically resulting in the release of Fe the sediment and will likely increase the extent of eutrophication in aquatic environments at higher temperatures.

## Supporting Information

Figure S1 **Phosphorus dynamics in unamended(a), and cyanobacterial bloom biomass (CBB)-amended sediments(b) at 32°C.**
(DOC)
