# Supplementary material for: Temperature and Cyanobacterial Bloom Biomass Influence Phosphorous Cycling in Eutrophic Lake Sediments
Source: PLoS One. 2014 Mar 28;9(3):e93130. doi: 10.1371/journal.pone.0093130 (PMC3969358; doi:10.1371/journal.pone.0093130)
Supplement: Figure S1 — Phosphorus dynamics in unamended(a), and cyanobacterial bloom biomass (CBB)-amended sediments(b) at 32°C. (DOC) [file pone.0093130.s001.doc]

**Temperature and cyanobacterial bloom biomass influence Phosphorous Cycling in Eutrophic Lake Sediments**

Mo Chen^a,b^, Tian-ran Ye^a^, Lee R. Krumholz^c^, He-Long Jiang^a^*

^a^State Key Laboratory of Lake Science and Environment, Nanjing Institute of Geography and Limnology, Chinese Academy of Sciences, Nanjing 210008, China

^b^Graduate University of Chinese Academy of Sciences

^c^Department of Microbiology and Plant Biology, University of Oklahoma, Norman, Oklahoma, USA

^*^Corresponding author. Mail address: Nanjing Institute of Geography and Limnology, Chinese Academy of Sciences, 73 East Beijing Road, Nanjing 210008, China. Tel. /fax: 0086-25-8688 2208. E-mail: [hljiang@niglas.ac.cn](mailto:hljiang@niglas.ac.cn) (H.L. Jiang).

**(a) No cyanobacterial biomass amendment (b) Cyanobacterial biomass amendment**

**
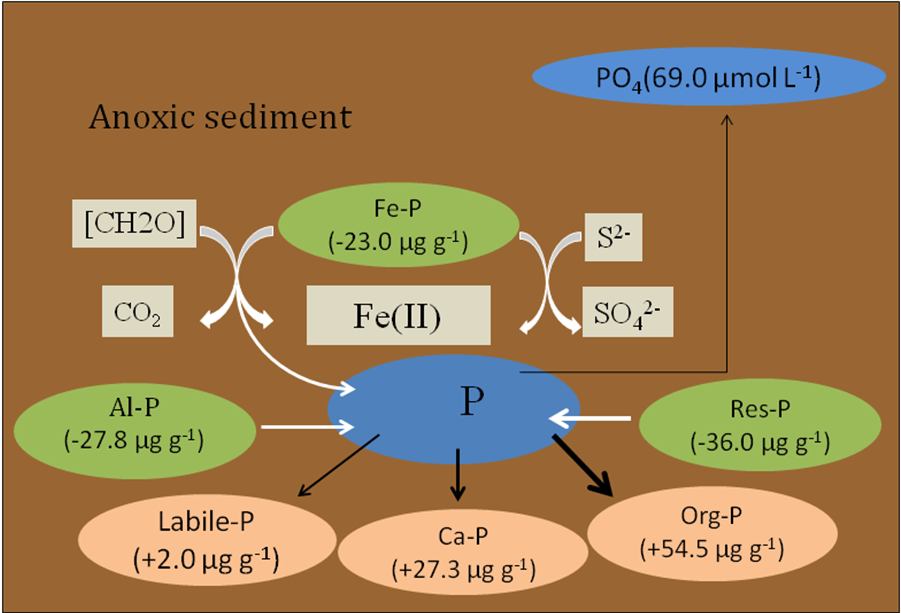
**
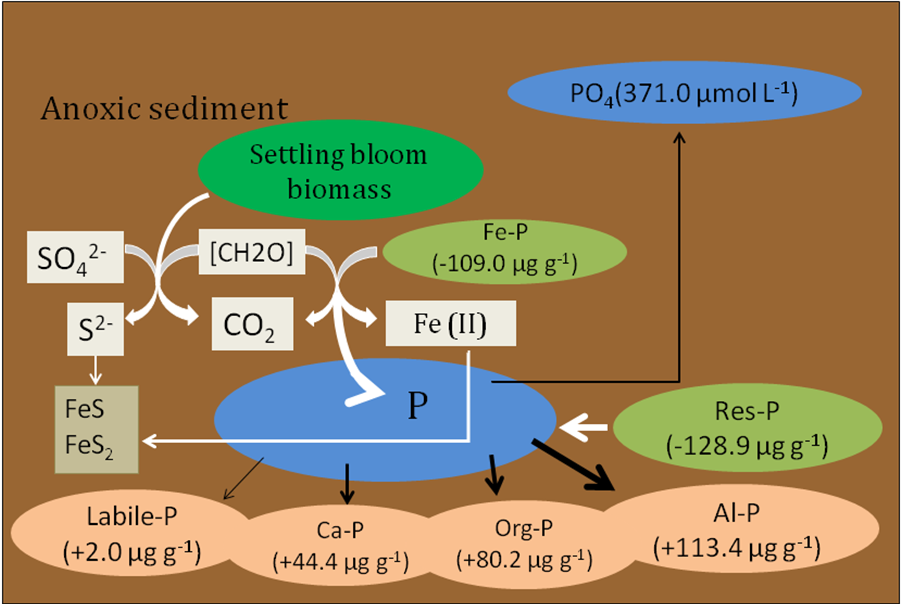


**Figure S1** Phosphorus dynamics in unamended (a), and cyanobacterial bloom biomass (CBB)-amended sediments (b) at 32°C.
